# SMAD4 Expression and Its Association With Treatment Resistance and Clinical Outcomes in Oral Squamous Cell Carcinoma Treated With Preoperative Chemoradiotherapy

**DOI:** 10.1002/cnr2.70654

**Published:** 2026-08-11

**Authors:** Junki Sakata, Akiyuki Hirosue, Ryoji Yoshida, Yuichiro Matsuoka, Yusei Todoroki, Hidetaka Arita, Hikaru Nakashima, Tatsuro Yamamoto, Nozomu Takahashi, Masatoshi Hirayama, Kenta Kawahara, Ryo Toya, Ryuji Murakami, Takaaki Ito, Yoshikazu Kuwahara, Hideki Nakayama

**Affiliations:** ^1^ Department of Oral and Maxillofacial Surgery, Faculty of Life Sciences Kumamoto University Kumamoto Japan; ^2^ Department of Oral and Maxillofacial Surgery Jichi Medical University Tochigi Japan; ^3^ Division of Oral and Maxillofacial Surgery Tsuruta Hospital Kumamoto Japan; ^4^ Section of Oral Oncology Fukuoka Dental College Fukuoka Japan; ^5^ Division of Oral and Maxillofacial Surgery Ariake Medical Center Arao Japan; ^6^ Department of Radiological Sciences, Graduate School of Biomedical Sciences Nagasaki University Nagasaki Japan; ^7^ Department of Medical Imaging, Faculty of Life Sciences Kumamoto University Kumamoto Japan; ^8^ Kumamoto Health Science University Kumamoto Japan; ^9^ Radiation Biology and Medicine, Faculty of Medicine Tohoku Medical and Pharmaceutical University Sendai Japan

**Keywords:** apoptosis, chemoresistance, oral squamous cell carcinoma, SMAD4, therapeutic resistance

## Abstract

**Background:**

Small mothers against decapentaplegic homologue 4 (SMAD4) plays an important role in transforming growth factor (TGF)‐β signaling and is associated with various cancers. However, little is known about SMAD4 expression in oral squamous cell carcinoma (OSCC).

**Aim:**

To investigate the clinical and biological significance of SMAD4 expression in OSCC.

**Methods and Results:**

Immunohistochemical analysis was performed to detect SMAD4 expression in 90 OSCC specimens obtained from patients who underwent preoperative chemoradiotherapy and surgery (phase II study). We also investigated the correlation between SMAD4 expression and various clinicopathological features. In vitro experiments were performed to assess the effects of SMAD4 expression on chemosensitivity and changes in apoptosis‐related molecules. Notably, low SMAD4 expression was significantly correlated with an increased risk of lymph node metastasis, poor response to chemoradiotherapy, and adverse prognosis in patients with OSCC who underwent preoperative 5‐FU‐based chemoradiotherapy. Additionally, SMAD4 downregulation in OSCC cells decreased sensitivity to cisplatin and 5‐fluorouracil and was associated with an increase in cIAP2 expression.

**Conclusion:**

The reduced expression of SMAD4 has been linked to both chemotherapy resistance and prognosis in OSCC. However, it may not function as an independent prognostic biomarker. Further research is necessary to determine whether these findings can be generalized to the broader OSCC population.

## 1 Introduction

Oral squamous cell carcinoma (OSCC) is a common malignancy of the oral cavity, especially eastern Aisa including Japan [1]. Despite the development of diagnostic techniques and multidisciplinary treatment methods that combine surgery, chemotherapy, radiotherapy, and immunotherapy, there has been no dramatic change in the prognosis of patients with OSCC [2]. Chemotherapy is often employed as part of a multimodal therapeutic strategy for OSCC. However, the development of chemotherapy resistance remains a significant clinical challenge. Therefore, elucidating the molecular mechanisms underlying chemoresistance in OSCC is essential for developing effective therapeutic strategies.

Based on our current understanding, multiple factors, including apoptosis, drug inactivation, and DNA damage repair, contribute to innate and acquired chemoresistance in OSCC [3]. Apoptosis is a physiological process that leads to cell death, with caspases acting as central regulators. Caspases are classified into two main categories: initiator caspases (caspase‐8, ‐9, and ‐10) and effector caspases (caspases‐3, ‐6, and ‐7). Initiator caspases cleave downstream effector caspases, which then cleave cytoskeletal and nuclear proteins, such as poly (ADP‐ribose) polymerase (PARP), to induce apoptosis [4]. Inhibitor of apoptosis proteins (IAPs), including cellular IAP1 (cIAP1), cellular IAP2 (cIAP2), X‐linked IAP (XIAP), and survivin, also regulate apoptosis by directly inhibiting caspases [5, 6], thereby contributing to cancer cell chemoresistance [7, 8].

Small mothers against decapentaplegic homologue 4 (SMAD4) has been identified as a tumor suppressor in pancreatic cancer. It plays a critical role in regulating transforming growth factor (TGF)‐β signaling [9, 10]. SMAD4 forms a trimeric complex with receptor‐activated SMADs (SMAD2 and 3 or SMAD1 and 5) and facilitates the nuclear translocation of the SMAD complex, where it binds to the SMAD‐binding element of target genes involved in cancer‐related processes [11]. Although loss of SMAD4 is crucial for the malignant progression of several cancers [12, 13], its clinical significance in OSCC remains unclear. To date, there are no data on the relationship between SMAD4 and the IAP family, although the regulation of XIAP expression via TGF‐β signaling has been reported [14]. Therefore, the possibility that SMAD4, a key regulator of TGF‐β signaling, affects IAP family expression warrants investigation. Accordingly, we focused on variations in the expression of SMAD4 and anti‐apoptotic molecules, such as IAPs.

In this study, we evaluated SMAD4 protein expression in biopsy specimens obtained from patients with OSCC. Specifically, we determined whether low SMAD4 expression correlated with poor response to preoperative chemoradiotherapy. In addition, we conducted an in vitro analysis to investigate the impact of SMAD4 expression on cell death induced by anticancer drugs and radiation, and its association with the expression of apoptosis‐related molecules.

## 2 Materials and Methods

### 2.1 Clinical Samples and Ethical Considerations

This study was conducted as a retrospective analysis involving 90 patients with advanced oral squamous cell carcinoma (OSCC) who were treated at Kumamoto University Hospital between October 2003 and June 2011. All patients had previously participated in a phase II study and received preoperative 5‐fluorouracil (5‐FU)‐based chemoradiotherapy (CRT) followed by radical surgery [15, 16]. The study utilized archived residual tissue samples that were originally collected during routine clinical procedures for histopathological diagnosis. These included biopsy specimens obtained before CRT for immunohistochemical staining and postoperative radical resection specimens used to determine pathological responses and observe changes in residual tumors. The research protocol followed the guidelines of the Kumamoto University Ethics Committee and was approved in 2017 (RINRI No. 1427) to specifically authorize the retrospective use of these historical clinical samples. This approval was granted under the overarching protocol title, “Explore molecules involved in the development and progression of oral cancer” which encompasses the present research. Accordingly, while the approval letter reflects this broad protocol name, the specific title of this manuscript was defined to focus on SMAD4 expression. Importantly, all research activities, including immunohistochemical staining and data analysis, were strictly performed after obtaining this ethical approval. In accordance with ethical guidelines, study information was disclosed on the institution's website to provide patients with an opt‐out opportunity. The inclusion criteria were: (1) pathologically confirmed OSCC and (2) completion of the prescribed preoperative CRT protocol. Patients were excluded if they had: (1) a previous history of head and neck radiotherapy or chemotherapy or (2) the presence of multiple primary cancers. The sample size (*n* = 90) was determined by including all consecutive patients who met these criteria during the study period to minimize selection bias. Tumors were staged according to the eighth edition of the AJCC TNM classification [17], and the degree of differentiation was determined using the World Health Organization grade classification [18]. Histological responses to CRT were evaluated based on the Shimosato criteria a grade of ≤IIa was defined as a poor response to treatment [19]. Detailed clinical data are provided as Table S1.

### 2.2 Immunohistochemical Staining and Assessment

Immunohistochemistry was performed to validate the expression of specific proteins in OSCC tissue sections, as previously described [15]. For negative controls, the primary antibody was replaced with phosphate‐buffered saline to confirm the absence of non‐specific staining. Three observers, unaware of the patients' clinical status, evaluated the immunoreactivity of the sections for SMAD4 expression. Immunostaining was performed as previously described [20]. Each sample was scored based on the intensity and percentage of stained cells, and the combined score was used as the final immunostaining score. A score ≥ 5 indicated high SMAD4 expression.

### 2.3 Cell Lines and Cell Culture

Human OSCC cell lines, SAS and HSC‐2, were cultured in Dulbecco's Modified Eagle's medium (DMEM) containing 10% fetal bovine serum (FBS). All cell lines were purchased from the Japanese Collection of Research Bioresources Cell Bank of the National Institute for Biomedical Innovation, Health and Nutrition (Osaka, Japan). The cell lines underwent short tandem repeat (STR) analysis immediately upon acquisition, employing the methodology described by Yamana et al. [21]. Cells at 2–3 passages post‐purchase were utilized for experimental procedures. Furthermore, mycoplasma contamination was assessed through Hoechst staining and verified with the mycoplasma detection kit (MycoStrip, InvivoGen, San Diego, CA, USA), ensuring the absence of contamination prior to use.

### 2.4 Small Interfering RNA (siRNA) Transfection

siRNA transfection experiments were conducted as previously described [22]. Cells were transfected with either 20 nM of Stealth siRNA specifically designed to target SMAD4 or an equal concentration of non‐targeting negative control siRNA (Stealth RNAi Negative Control; Invitrogen, Waltham, MA, USA) using Lipofectamine RNAiMAX (Invitrogen) according to the manufacturer's instructions. This negative control was used to ensure that the observed biological effects were specific to SMAD4 silencing and not a result of the transfection procedure or non‐specific RNA interference. After 24 h of transfection, the cells were collected for further experiments.

### 2.5 RNA Isolation and Quantitative Real‐Time PCR (qRT–PCR)

Total RNA was purified using a mirVana miRNA Isolation Kit (Life Technologies, Palo Alto, CA, USA) and reverse‐transcribed to generate cDNA using a ReverTra qPCR RT Kit (Toyobo, Osaka, Japan). Each PCR was performed on a LightCycler 1.5 (Roche, Indianapolis, IN, USA) using Thunderbird SYBR qPCR Mix (Toyobo). Thereafter, the data obtained from qRT‐PCR were analyzed using the 2^−ΔΔCt^ method on a CFX Connect system (Bio‐Rad, Hercules, CA, USA), with *GAPDH* as an internal control. The primer sets used are listed in Table S2. All experiments were performed in triplicates.

### 2.6 Western Blotting

Western blotting was performed as previously described [22]. The following antibodies were employed to detect the protein of interest: SMAD4 (1:200, H‐552, Santa Cruz), cIAP1 (1:200, H‐85, Santa Cruz), cIAP2 (1:200, H‐83, Santa Cruz), XIAP (1:1000, Cell Signaling Technology, Danvers, MA, USA), Survivin (1:1000, Cell Signaling Technology) and β‐actin (1:10000, Sigma, St. Louis, MO, USA). β‐actin served as the internal control. The secondary antibodies employed included HRP‐conjugated anti‐rabbit IgG at a dilution of 1:10000 and HRP‐conjugated anti‐mouse IgG at a dilution of 1:15000, both sourced from Cell Signaling Technology. Protein bands were detected using an enhanced chemiluminescence system.

### 2.7 Drug Sensitivity Assay

A drug sensitivity assay was performed as previously described [23]. Briefly, the cells were seeded into 96‐well plates and incubated in DMEM containing 10% FBS for 24 h. Thereafter, DMEM containing various concentrations of cisplatin or 5‐FU was added to each well, and cell viability was determined using the WST‐8 reagent (Cell Counting Kit‐8; Dojindo). Finally, the absorbance at 450 nm was measured using a microplate reader (model 680; Bio‐Rad). The experiments were conducted in triplicate, and the half‐maximal inhibitory concentrations (IC_50_) were calculated based on the survival curve. Cells transfected with non‐targeting negative control siRNA (Stealth RNAi Negative Control; Invitrogen, Waltham, MA, USA) were used as controls in the experiments. All experiments were performed in triplicates.

### 2.8 Irradiation

Irradiation was performed using a 150 KVp X‐ray generator with total filtration through a 0.5 mm aluminum plus 0.1 mm copper filter (MBR‐1520R; Hitachi). The dose rate (1.01 Gy per min) was measured using a thimble ionization chamber (IC 17A; Far West Technology). Cells transfected with non‐targeting negative control siRNA (Stealth RNAi Negative Control; Invitrogen, Waltham, MA, USA) were used as controls in the experiments. All experiments were performed in triplicates.

### 2.9 Modified High‐Density Survival (mHDS) Assay

We have utilized the mHDS assay to assess radiation‐induced cell death [23, 24]. This approach is advantageous because, by allowing cells to be cultured for an extended duration post‐irradiation, the mHDS assay can thoroughly evaluate all cytotoxic responses. It encompasses not only early cell death mechanisms, such as apoptosis and necrosis, but also late cell death processes, including cellular senescence and mitotic death, which manifest several days later. Furthermore, while cell density may introduce bias in colony formation assays, the mHDS assay employs high‐density cell cultures, thereby mitigating issues such as delayed cell growth and is considered to more accurately reflect clinically relevant, in vivo–like phenotypes. An overview of the method is provided below. Briefly, the cells transfected with control or SMAD4‐specific siRNA were irradiated at single doses of 5 and 10 Gy and incubated for 72 h. Thereafter, 10% of the cells in each flask were seeded in a 60‐mm culture dish and incubated for an additional 72 h. Cell numbers were determined via trypan blue exclusion, and a cell survival graph was plotted. The survival rate of cells at each administered dose was determined by comparing the final total cell count to that of the control group transfected with non‐targeting negative control siRNA (Stealth RNAi Negative Control; Invitrogen, Waltham, MA, USA). All experiments were performed in triplicates. Based on our previous research results, in vitro experiments using OSCC, the number of surviving cells significantly decreases with IR doses of 4 Gy or higher, and the presence or absence of radioresistance is most clearly reflected in experimental results at doses of 5 Gy or higher [25]. Therefore, in this study, the irradiation doses were set at 5 and 10 Gy.

### 2.10 Densitometric Analysis

The intensity of the protein bands was quantified using ImageJ software (National Institutes of Health, Bethesda, MD, USA). The signal intensity of each target protein was normalized to that of the internal control (β‐actin) from the same blot. The data are presented as the fold change relative to the control group, which either received no treatment or was transfected with non‐targeting negative control siRNA, depending on the situation.

### 2.11 Statistical Analysis

Fisher's exact test was used to determine the association between SMAD4 expression in tissue specimens and clinical characteristics. The Kaplan–Meier method was used for survival analysis, and the log‐rank test was used to assess the correlation between SMAD4 expression and patient survival. Additionally, a Cox regression model was used for multivariate survival analysis to study the effects of SMAD4 expression on overall survival (OS) and disease‐free survival (DFS). In vitro experiments were independently repeated at least three times using separately cultured cells to ensure reproducibility. For each independent experiment, measurements were performed in quintuplicate, and the averaged values were used for statistical analysis. Comparisons between two groups were performed using the student's *t*‐test, while comparisons among multiple groups were conducted using one‐way analysis of variance (ANOVA) followed by Dunnett's post hoc test. Statistical significance was set at *p* < 0.05. All analyses were conducted using the JMP 9 software (SAS Institute Inc., Cary, NC, USA).

## 3 Results

### 3.1 Clinical Significance of SMAD4 Expression in Patients With OSCC

**FIGURE 1:**
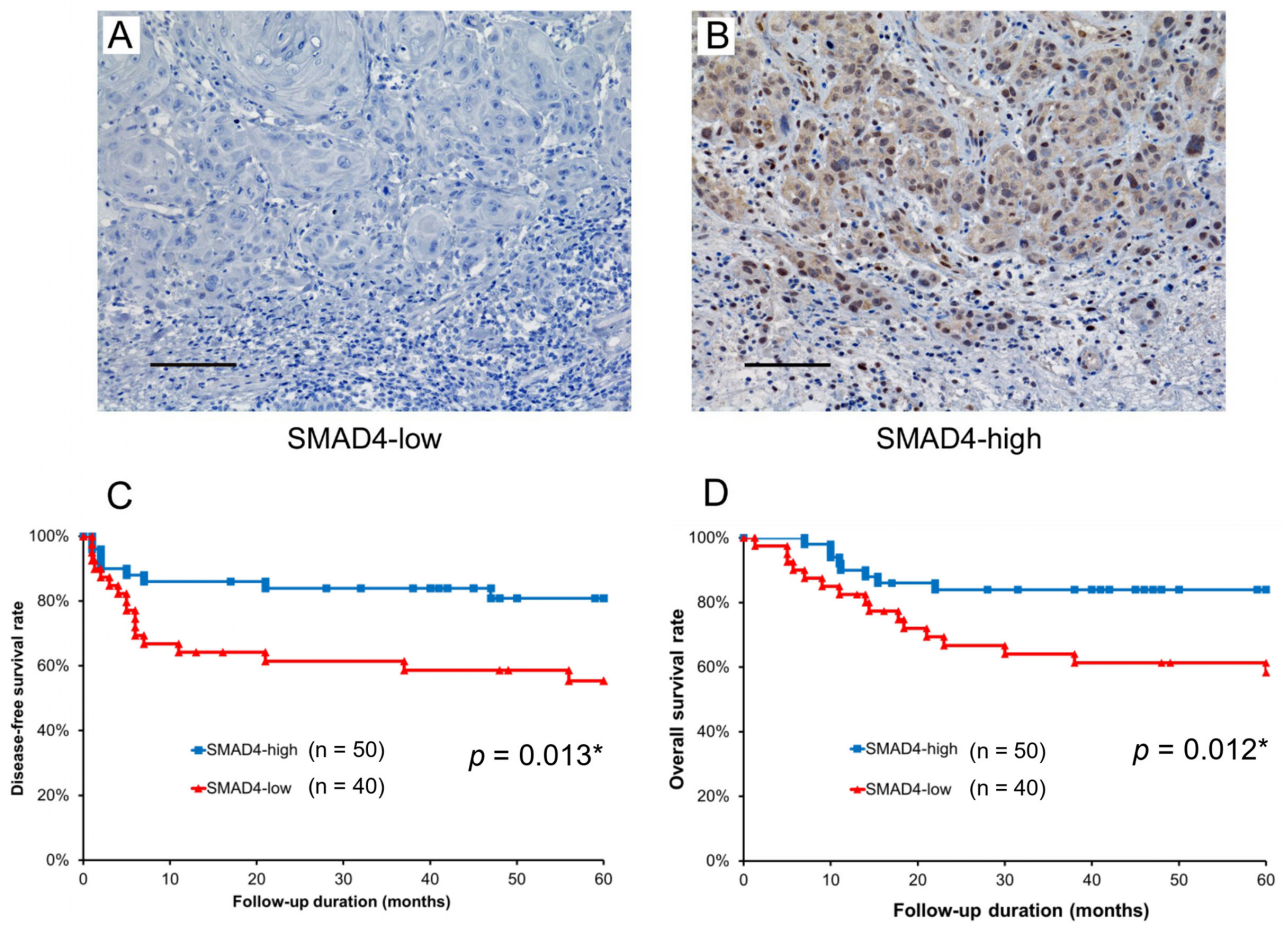
SMAD4 expression status affects the survival of patients with oral squamous cell carcinoma (OSCC). (A, B) Immunohistochemical staining to detect SMAD4 in OSCC biopsy specimens. Representative microscopic images show the characteristic expression status (A: Low expression; B: High expression). Scale bar, 100 μm. (C, D) Kaplan–Meier curves of (C) overall survival (OS) and (D) disease‐free survival (DFS) in patients with OSCC based on their SMAD4 expression status. *, *p* < 0.05.

To investigate the clinical and biological significance of SMAD4 expression in OSCC, we performed immunohistochemical analyses of biopsy samples from 90 patients with OSCC. SMAD4 expression was primarily localized to the nucleus and weakly localized to the cytoplasm of epithelial cells, with a heterogeneous expression pattern (Figure 1A,B). Based on immunostaining scores, patients were classified into low or high SMAD4 expression groups. Among the 90 patients, 50 (55.6%) had high SMAD4 expression and 40 (44.4%) had low SMAD4 expression. As shown in Table 1, low SMAD4 expression was associated with a high incidence of positive lymph node metastasis (*p* = 0.014) and poor response to preoperative chemoradiotherapy (*p* = 0.005). No significant differences in SMAD4 expression were observed with respect to age, sex, primary site, T stage, pathological stage, or differentiation.

**TABLE 1:** Correlation between SMAD4 expression and clinicopathological factors in 90 patients with OSCC who underwent preoperative CRT.

Characteristics	Total	SMAD4 expression		*p*
		High; *n* (%)	Low; *n* (%)	
	90	50 (55.6)	40 (44.4)	
Age, years				
Median	69	67	70	
Range	30–86	30–86	36–82	
Under 65	34	23 (67.6)	11 (32.4)	0.072
Over 65	56	27 (48.2)	29 (51.8)	
Sex				
Male	51	32 (62.7)	19 (37.3)	0.116
Female	39	18 (46.2)	21 (53.8)	
Primary site				
Tongue	21	15 (71.4)	6 (28.6)	0.645
Mandible	27	14 (51.9)	13 (48.1)	
Maxilla	15	8 (53.3)	7 (46.7)	
Buccal mucosa	15	8 (53.3)	7 (46.7)	
Oral floor	10	4 (40.0)	6 (60.0)	
Palate	2	1 (50.0)	1 (50.0)	
pT‐stage				
T1, T2	36	19 (52.8)	17 (47.2)	0.923
T3	19	12 (63.2)	7 (36.8)	
T4	35	19 (54.3)	16 (45.7)	
pN‐stage				
N0	49	33 (67.3)	16 (32.7)	0.014*
≥ N1	41	17 (41.5)	24 (58.5)	
Pathological stage				
II	23	16 (69.6)	7 (30.4)	0.289
III	25	13 (52.0)	12 (48.0)	
IV	42	21 (50.0)	21 (50.0)	
Differentiation				
Well	67	40 (59.7)	27 (40.3)	0.097
Moderate	21	8 (38.1)	13 (61.9)	
Poor	2	2 (100)	0 (0)	
Pathological response				
0, I, IIa	14	3 (21.4)	11 (78.6)	0.005**
IIb, III, IV	76	47 (61.8)	29 (38.2)	

*Note:* Fisher's exact test was used to examine the correlation between SMAD4 expression and clinicopathological factors. **, *p* < 0.01, *, *p* < 0.05.

Abbreviations: CRT, chemoradiotherapy; OSCC, oral squamous cell carcinoma.

### 3.2 Relationships Between SMAD4 Expression and Survival of Patients With OSCC

To determine the prognostic value of SMAD4 expression in patients with OSCC, univariate and multivariate analyses were performed using the Kaplan–Meier method and Cox proportional hazard regression models. Utilizing the Kaplan–Meier method, it was determined that the prognosis was significantly poorer in the group with low SMAD4 expression (DFS; *p* = 0.013, Figure 1C, OS; *p* = 0.012, Figure 1D). Furthermore, the presence of positive lymph node metastasis (≥ N1, DFS; *p* < 0.001, OS; *p* < 0.001) (Figure S1A,B) and a poor response to preoperative chemoradiotherapy (≤ IIa) (≤ IIa, DFS; *p* < 0.001, OS; *p* < 0.001) (Figure S1C,D) were significantly correlated with an unfavorable prognosis for both DFS and OS. In the multivariate Cox regression analysis, lymph node metastasis and the response to preoperative chemoradiotherapy were identified as independent prognostic factors for disease‐free survival (DFS), with lymph node metastasis showing a hazard ratio (HR) of 3.215 (95% CI, 1.36–8.450, *p* = 0.007) and response to preoperative chemoradiotherapy demonstrating an HR of 2.79 (95% CI, 1.083–6.476, *p* = 0.034). In terms of overall survival (OS), lymph node metastasis was the sole independent prognostic factor, with an HR of 3.142 (95% CI, 1.234–9.038, *p* = 0.016). The expression status of SMAD4 did not achieve statistical significance for either DFS or OS (Table 2).

**TABLE 2:** The results of the multivariate analysis of prognostic factors by the Cox proportional hazards regression model.

Characteristics	Assigned score	OS		DFS	
		Hazard ratio (95% CI)	*p*	Hazard ratio (95% CI)	*p*
SMAD4 expression					
Low	1	1.510 (0.573–4.098)	0.404	1.625 (0.673–4.075)	0.281
High	0				
pN‐stage					
N0	0	3.142 (1.234–9.038)	0.016*	3.215 (1.362–8.450)	0.007**
≥ N1	1				
Pathological response					
0, I, IIa	1	2.509 (0.953–6.462)	0.062	2.709 (1.083–6.476)	0.034*
IIb, III, IV	0				

*Note:* Cox regression model was employed for multivariate survival analysis to study the effects of SMAD4 expression on patients' prognosis. *, *p* < 0.05, **, *p* < 0.01.

Abbreviations: CI, confidence interval; DFS, disease‐free survival; OS, overall survival.

### 3.3 Effect of SMAD4 on Chemosensitivity in OSCC Cells

**FIGURE 2:**
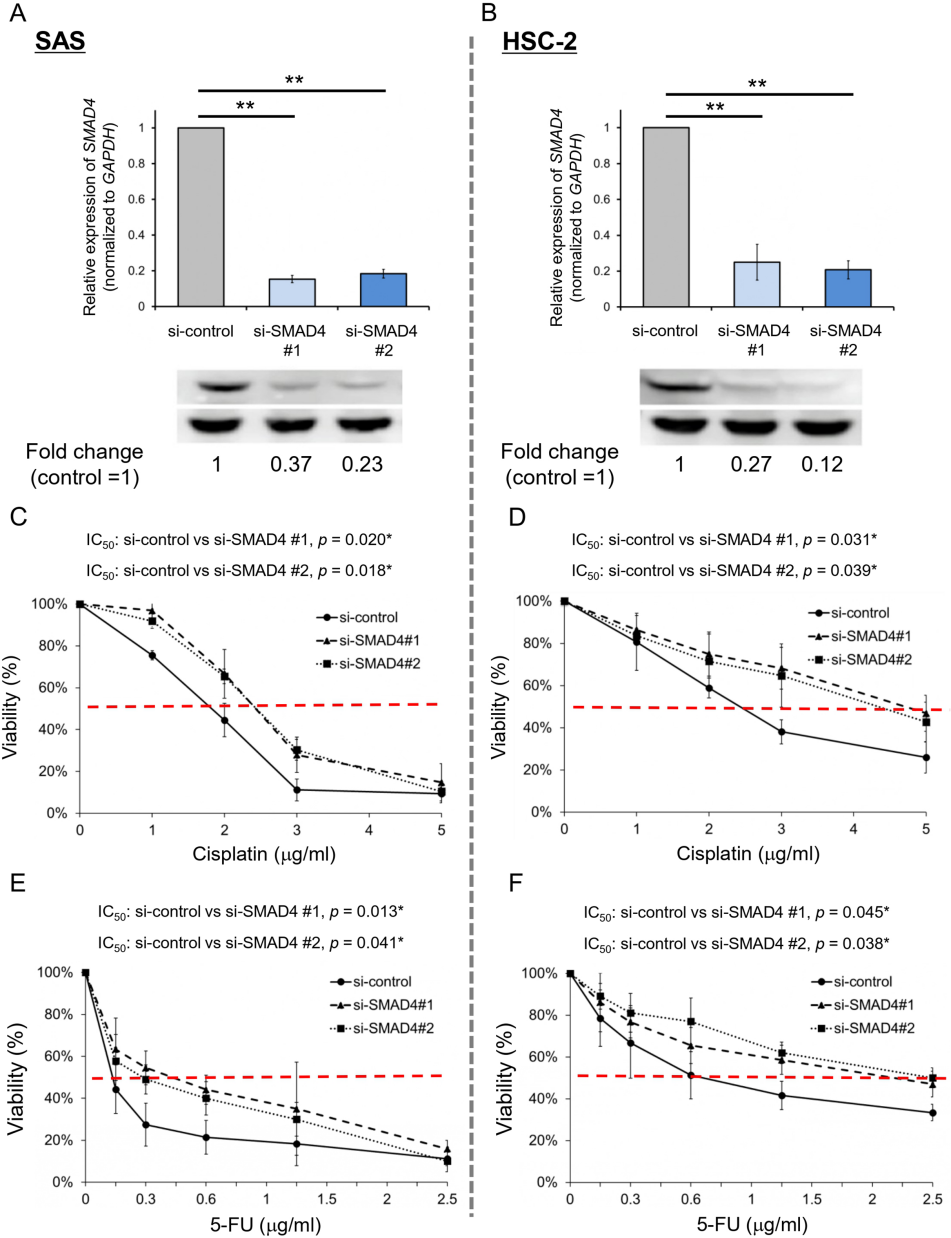
SMAD4 confers resistance to chemo‐drugs in oral squamous cell carcinoma cells. Quantitative RT‐PCR and western blotting were performed to examine SMAD4 mRNA and protein levels in SAS (A) and HSC‐2 cells (B) after transfection with control or SMAD‐specific siRNA. Relative expression of SMAD4 proteins was calculated by using image J software. SAS (C, D) and HSC‐2 cells (E, F) transfected with SMAD4 siRNA or control siRNA were treated with different concentrations of cisplatin (C, D) and 5‐fluorouracil (E, F) for 72 h. WST assay was used to quantify cell viability. The y‐axis of the graph indicates cell viability, while the x‐axis represents the concentration of the drug. The red dashed line indicates the IC_50_ level. Results are shown as the means ± standard deviation of three independent experiments. *, *p* < 0.05; **, *p* < 0.01.

Next, we investigated the effect of SMAD4 expression on the sensitivity of OSCC cells to chemotherapy and radiotherapy. To achieve this, we conducted chemosensitivity and mHDS assays using OSCC cells treated with SMAD4‐specific siRNA, followed by western blotting and qRT‐PCR to evaluate the changes in SMAD4 expression at the protein and mRNA levels. Transfection with SMAD4 siRNAs #1 and #2 significantly suppressed SMAD4 expression at both the protein and mRNA levels (Figure 2A,B). Notably, the chemosensitivity assay revealed that the sensitivity of OSCC cells to cisplatin and 5‐FU decreased significantly after transfection with SMAD4‐specific siRNA (Figure 2C–F). Furthermore, the IC_50_ values of cisplatin and 5‐FU were significantly higher against siRNA‐transfected OSCC cells (SAS and HSC‐2) than against control cells.

### 3.4 Effect of SMAD4 on Radiosensitivity in OSCC Cells

**FIGURE 3:**
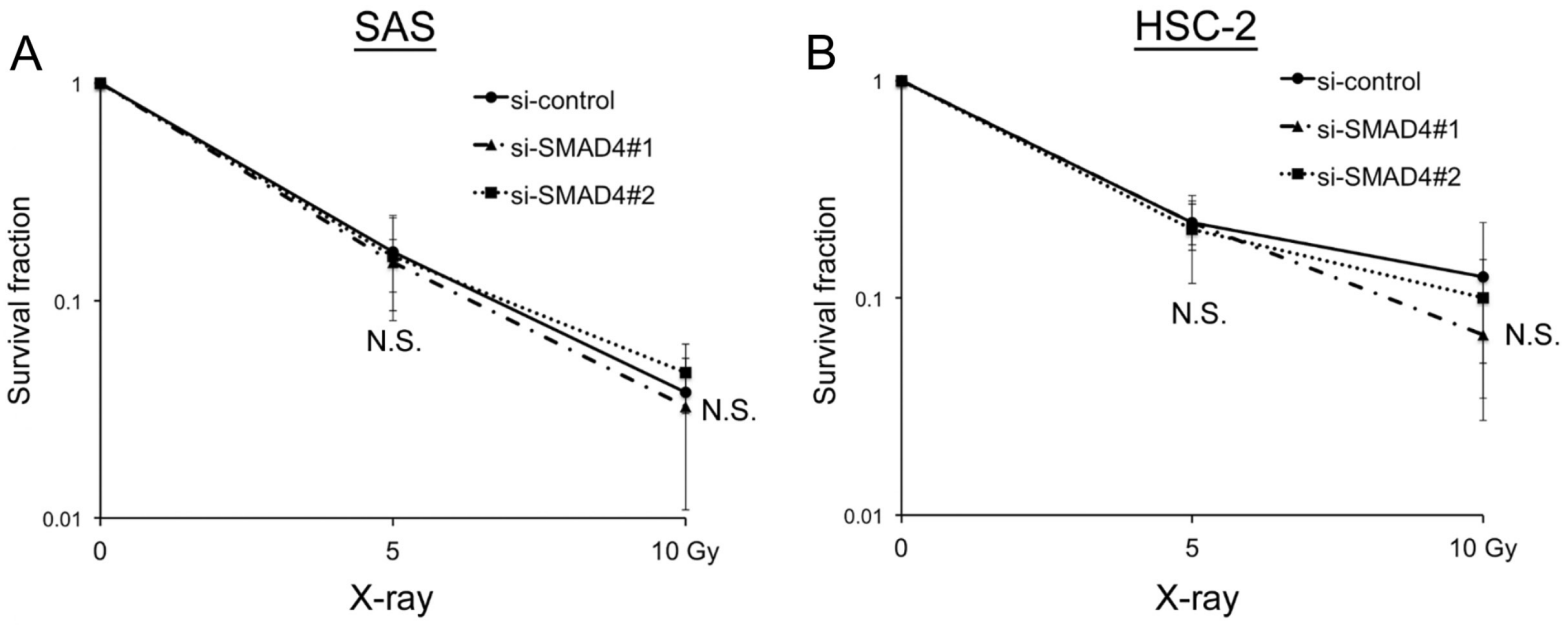
SMAD4 expression did not affect radiosensitivity in oral squamous cell carcinoma (OSCC) cells. (A) SAS and (B) HSC‐2 cells were transfected with SMAD4 siRNA or control siRNA and exposed to X‐ray doses of 0, 5, and 10 Gy. The surviving fraction was evaluated using a mHDS assay. The y‐axis of the graph shows cell viability on a logarithmic scale, while the x‐axis represents the doses of the X‐ray. Results are shown as the means ± standard deviation of three independent experiments. N.S., not significant.

Given the focus on SMAD4 and treatment resistance in clinical samples from patients with OSCC who have undergone CRT, clarifying the relationship between SMAD4 and chemotherapy sensitivity and radiosensitivity is crucial. Accordingly, we examined changes in the radiosensitivity of SAS and HSC‐2 cells following SMAD4 knockdown. Importantly, mHDS assay showed that SMAD4 knockdown did not affect the radiosensitivity of the two OSCC cell lines (Figure 3).

### 3.5 Relationships Between SMAD4 and IAPs in OSCC Cells

**FIGURE 4:**
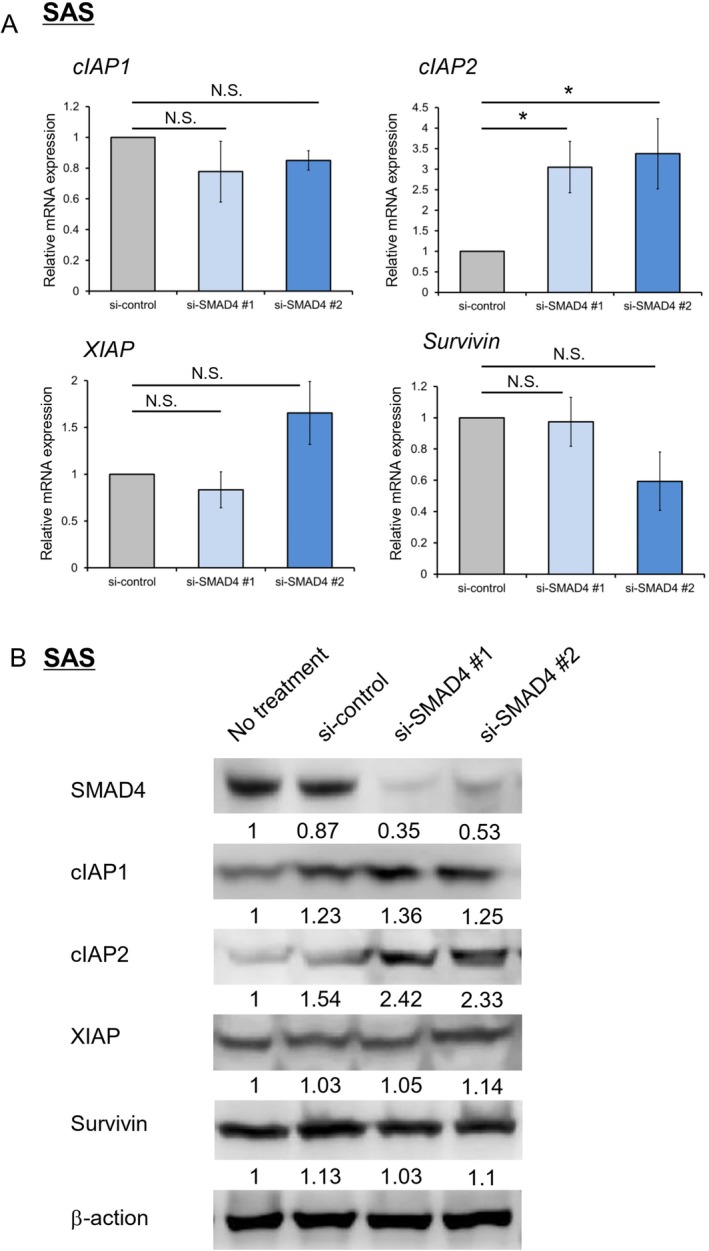
Expression of cIAP2 increased due to SMAD4 knockdown in SAS cells. (A) Quantitative RT‐PCR was performed to detect *cIAP1, cIAP2, XIAP* and Survivin mRNA levels in SAS cells transfected with SMAD4‐specific or control siRNA. The y‐axis of the graph indicates the relative mRNA expression levels normalized to the internal control, GAPDH. The x‐axis represents the type of siRNA. Results are shown as the means ± standard deviation of three independent experiments. N.S., not significant. *, *p* < 0.05. (B) Western blotting was performed to detect cIAP1, cIAP2, XIAP, Survivin, Bcl‐2, and Bcl‐xL at protein levels in SAS cells transfected with SMAD4 siRNA or control siRNA. The relative expression levels of each molecule were quantified using ImageJ software, as indicated below the target proteins. β‐Actin was used as internal control.

**FIGURE 5:**
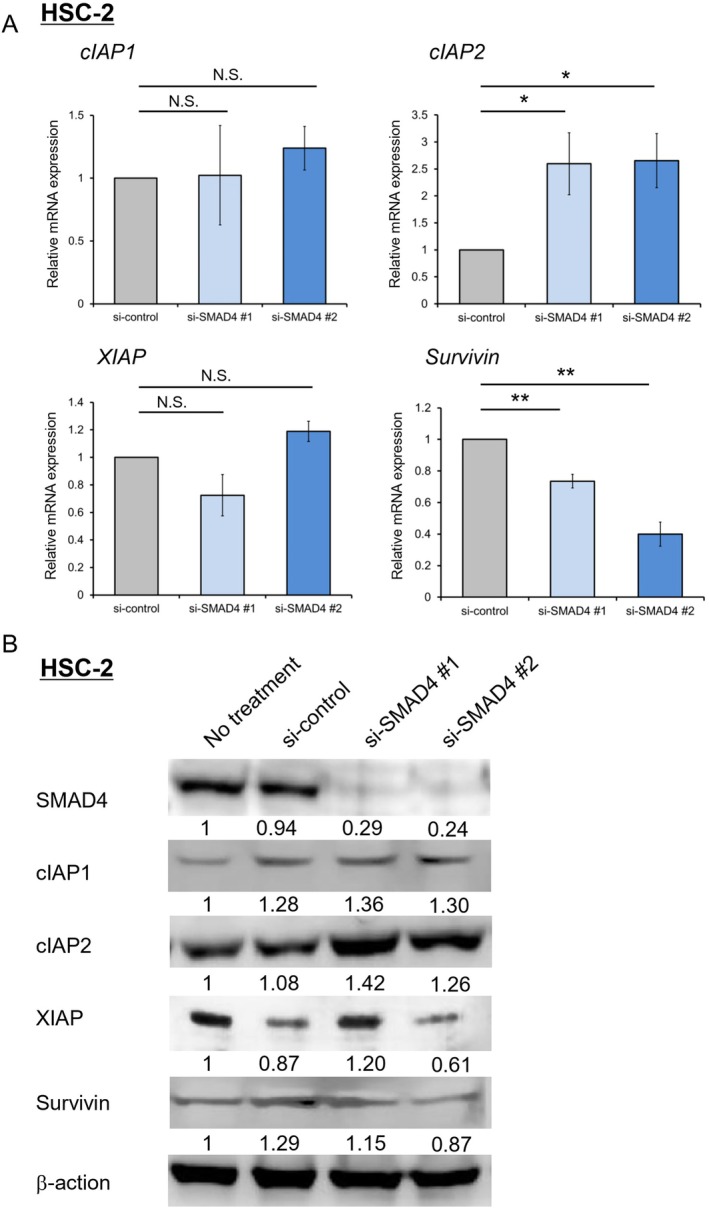
Expression of cIAP2 increased due to SMAD4 knockdown in HSC‐2 cells. (A) Quantitative RT‐PCR was performed to detect *cIAP1, cIAP2, XIAP* and Survivin mRNA levels in SAS cells transfected with SMAD4‐specific or control siRNA. The y‐axis of the graph indicates the relative mRNA expression levels normalized to the internal control, GAPDH. The x‐axis represents the type of siRNA. Results are shown as the means ± standard deviation of three independent experiments. N.S., not significant. *, *p* < 0.05. **, *p* < 0.01. (B) Western blotting was performed to detect cIAP1, cIAP2, XIAP, Survivin, Bcl‐2, and Bcl‐xL at protein levels in HSC‐2 cells transfected with SMAD4 siRNA or control siRNA. The relative expression levels of each molecule were quantified using ImageJ software, as indicated below the target proteins. β‐Actin was used as internal control.

To investigate the relationship between SMAD4 and IAPs, quantitative real‐time polymerase chain reaction (qRT‐PCR) and western blotting assays were carried out. In SAS cells, the transfection of SMAD4‐specific siRNA resulted in a notable upregulation of cIAP2 expression at the mRNA level (Figure 4A). Additionally, this upregulation of cIAP2 was corroborated at the protein level (Figure 4B). In HSC‐2 cells, the introduction of SMAD4‐specific siRNA similarly led to a significant increase in cIAP2 expression at the mRNA level; however, unlike in SAS cells, there was a marked decrease in Survivin expression (Figure 5A). At the protein level, as observed in SAS cells, there was a distinct increase in cIAP2 expression, although the decrease in Survivin expression was less pronounced (Figure 5B). Consequently, among SAS and HSC‐2 cells, cIAP2 was the sole molecule whose expression varied in a manner contingent upon SMAD4 expression. Furthermore, regarding XIAP, in HSC‐2 cells, a partial increase in expression was observed when SMAD4 expression was diminished (Figure 5B).

## 4 Discussion

This study revealed that SMAD4 expression is associated with chemoradiotherapy response and nodal status in patients with OSCC. Low SMAD4 expression was associated with poorer patient survival in the univariate analysis. Additionally, SMAD4 knockdown decreased the sensitivity of OSCC cells to cisplatin and 5‐FU, but not to irradiation. Moreover, SMAD4 knockdown was also associated with an increase in cIAP2 expression, which has anti‐apoptotic effects. These findings suggest that SMAD4 expression is associated with chemotherapy response, and its loss correlates with the altered expression of apoptosis‐related pathways. To the best of our knowledge, there are no existing studies that have examined the clinicopathological significance of SMAD4 expression in the primary lesion of OSCC in patients who have undergone preoperative chemoradiotherapy. Furthermore, there are no reports indicating that SMAD4 expression influences cIAP2 expression in OSCC cells. Consequently, this study may offer a potential avenue for further exploration into the biological significance of SMAD4 in OSCC.

Although SMAD4 has been shown to contribute to HNSCC carcinogenesis, including OSCC [20, 26], the relationship between SMAD4 expression and malignant OSCC phenotypes such as invasion, proliferation, and therapeutic resistance remains unclear. Moreover, although the relationship between SMAD expression and cetuximab sensitivity has been evaluated [27, 28], epithelial‐to‐mesenchymal transition (EMT) and MAPK/JNK activation have been linked to resistance acquisition but not directly to 5‐FU resistance. Furthermore, the clinical significance of SMAD4 expression in predicting the response to chemoradiotherapy in OSCC has not been well characterized. To address these gaps, we explored the biological role of SMAD4 in OSCC using samples from patients treated with 5‐FU‐based CRT. Immunohistochemical analysis revealed that SMAD4 expression was associated with therapeutic resistance, lymph node metastasis, and significantly poorer OS and DFS. However, in multivariate analysis, SMAD4 expression was not identified as an independent prognostic factor. These findings may be partially explained by the strong association between SMAD4 expression and other prognostic factors, such as lymph node metastasis and response to chemoradiotherapy. Thus, SMAD4 expression may reflect underlying biological processes that influence treatment response rather than acting as an independent prognostic determinant. In addition, since the analysis group in this study consisted of patients with OSCC who had undergone preoperative CRT, the clinicopathological significance of SMAD4 expression in patients receiving standard treatment still needs to be further investigated. Overall, we propose that SMAD4 expression in primary tumors could serve as a potential biomarker for treatment response and prognosis in patients with OSCC who underwent preoperative chemo−/chemoradiotherapy.

Based on the clinical analyses, we explored the impact of SMAD4 on chemoresistance and radioresistance in OSCC cells. Our in vitro experiments demonstrated that SMAD4 knockdown reduced the sensitivity of OSCC cells to cisplatin and 5‐FU. These results are consistent with previous studies showing that SMAD4 plays an important role in regulating chemotherapy sensitivity in several cancers. For example, SMAD4 has been identified as a predictive biomarker for 5‐FU‐based chemotherapy in colorectal cancer [29]. Additionally, SMAD4 expression enhances doxorubicin resistance in breast cancer [30]. Zhang et al. reported that loss of SMAD4 induced 5‐FU resistance and activated the Akt pathway, thereby upregulating the anti‐apoptotic proteins Bcl‐2, Bcl‐w, and survivin in colorectal cancer [31]. Ozawa et al. found that pro‐apoptotic pathways were downregulated in most SMAD4‐low head and neck cancers [28]. Our in vitro analyses may support these previous reports. Collectively, these findings support the hypothesis that SMAD4 regulates chemotherapy sensitivity through modulation of apoptosis‐related signaling pathways.

IAPs, which are endogenous caspase inhibitors, are expressed in cancer cells. Their overexpression is associated with chemoresistance and poor prognosis in various cancer types [32, 33, 34, 35]. A previous study showed that cIAP2 was overexpressed in 5‐FU‐resistant OSCC cell lines and that patients with cIAP2‐positive OSCC had lower survival rates and poor response to chemoradiotherapy [16]. Consistent with these findings, our results demonstrated that SMAD4 knockdown significantly increased cIAP2 expression in OSCC cells. These findings suggest that the concurrent increase in cIAP2 expression following SMAD4 loss is associated with chemotherapy resistance. In this study, SMAD4 expression was associated with cIAP2 expression, suggesting that the chemoresistance observed in SMAD4‐knockdown cells concurred with cIAP2 upregulation. IAPs are regulated at the transcriptional, translational, and post‐translational levels [36, 37, 38]. In the context of SMAD4's regulation of IAP family expression, there exists the potential for direct transcriptional activation, wherein Smad4 directly interacts with promoter regions, such as those of XIAP and c‐IAP2, to stimulate their expression. Alternatively, Smad4 may form complexes with other transcription factors, such as Sp1 or AP‐1, thereby synergistically enhancing the transcription of IAPs. Conversely, the observed differences in expression variability between cell lines for XIAP and Survivin may be attributed to the fact that, although SAS cells possess a mutation in p53, its function remains intact, whereas HSC‐2 cells exhibit a loss‐of‐function mutation in p53 [39]. Regardless, the potential for SMAD4 to regulate the expression of the IAPs family, as suggested by this study, is a significant topic that warrants further investigation and clarification using more advanced molecular biological techniques in the future. Especially, the experimental system that specifically regulates the expression of cIAP2 is considered to be useful.

In the present study, no discernible difference in radiosensitivity was observed between SMAD4 knockdown cells and control cells. However, reports indicate that, in certain cancer types, SMAD4‐dependent reactive oxygen species (ROS) production, radiation‐induced autophagy, and transcriptional regulation by circular RNA (circRNA) significantly influence radiosensitivity [40, 41]. Radiation‐induced cell death can occur through multiple mechanisms, including apoptosis, necroptosis, and autophagy [42, 43, 44]. Although further investigations are needed to confirm the relationship between SMAD4 expression and other cell death pathways, the limited effect of SMAD4 on radiosensitivity observed in this study may reflect the involvement of alternative cell death pathways that are independent of SMAD4‐mediated apoptosis. Further investigation is necessary to elucidate the relationship between SMAD4 and radioresistance in OSCC. Specifically, additional research is required to clarify the associations with various forms of cell death, considering the interactions with the diverse signaling pathways that engage in crosstalk.

Our findings indicate that low SMAD4 expression is associated with lymph node metastasis in patients with OSCC, suggesting that SMAD4 is involved in metastasis and treatment resistance in OSCC. Cancer metastasis significantly contributes to treatment failure and mortality in patients with malignant tumors [45]. In colorectal cancer, SMAD4 deficiency induces migration, invasion, angiogenesis, and metastasis, and upregulates VEGF expression [31, 46]. In addition, SIRT7‐mediated SMAD4 destabilization has been reported to promote migration and invasion in breast and oral cancers [47, 48]. Furthermore, a high degree of lymphocytic infiltration was observed in the subepithelial stroma of SMAD4‐low‐expressing epithelial cells, resulting in an altered microenvironment in mice model [26]. Overall, various mechanisms may be involved in lymph node metastasis associated with low SMAD4 expression, including vascularization, EMT‐induced acquisition of invasive and metastatic potential, and regulation of the tumor microenvironment. These studies suggest that SMAD4 may influence tumor progression through multiple mechanisms, including regulation of tumor cell invasion and modulation of the tumor microenvironment. Further studies are needed to examine the molecular mechanisms underlying metastasis induced by SMAD4 depletion in OSCC cells.

In conclusion, this study provides new insights into the relationship between SMAD4 expression, clinical significance, and apoptosis‐related pathways in OSCC. Our findings suggest that reduced SMAD4 expression is associated with chemotherapy resistance and a concomitant increase in the antiapoptotic factor cIAP2. The evaluation of SMAD4 expression in pretreatment biopsy specimens may hold clinical significance in forecasting responses to chemoradiotherapy and in determining patient prognosis, particularly for those who have undergone preoperative therapy. However, further studies are warranted to clarify the molecular mechanisms underlying SMAD4‐mediated chemoresistance and to explore potential therapeutic strategies targeting this pathway.

## Author Contributions

**Junki Sakata:** investigation, funding acquisition. **Akiyuki Hirosue:** investigation, data curation, methodology. **Ryoji Yoshida:** data curation, writing – original draft, writing – review and editing, validation, visualization, conceptualization, project administration. **Yuichiro Matsuoka:** validation, formal analysis, methodology. **Yusei Todoroki:** formal analysis. **Hidetaka Arita:** resources, software. **Hikaru Nakashima:** resources. **Tatsuro Yamamoto:** resources. **Nozomu Takahashi:** data curation. **Masatoshi Hirayama:** data curation. **Kenta Kawahara:** formal analysis. **Ryo Toya:** writing – review and editing, resources. **Ryuji Murakami:** resources, writing – review and editing. **Takaaki Ito:** conceptualization, supervision. **Yoshikazu Kuwahara:** supervision. **Hideki Nakayama:** conceptualization, project administration, writing – review and editing.

## Funding

This study was financially supported by JSPS KAKENHI through Grants JP19K21413 and JP20K18700.

## Ethics Statement

This study was conducted in accordance with the principles of the Declaration of Helsinki and was approved by the Ethics Committee of Kumamoto University (RINRI No. 1427).

## Consent

Informed consent was obtained from all the participants.

## Conflicts of Interest

The authors declare no conflicts of interest.

## Supporting information


**Figure S1:** Nodal metastasis and response to chemoradiotherapy are related to survival in patients with oral squamous cell carcinoma (OSCC). Kaplan–Meier curves of (A) overall survival (OS) and (B) disease‐free survival (DFS) in patients with OSCC based on pN‐stage, and (C) OS and (D) DFS in patients with OSCC based on the pathological response to chemoradiotherapy. **, *p* < 0.01.

**Table S1:** Clinicopathological and clinical outocome of patients with OSCC who included in this study.

**Table S2:** List of primers used for real‐time PCR.

## Data Availability

The data supporting the findings of this study are available from the corresponding author upon reasonable requests.

## References

[1] H. Sung , J. Ferlay , R. L. Siegel , et al., “Global Cancer Statistics 2020: GLOBOCAN Estimates of Incidence and Mortality Worldwide for 36 Cancers in 185 Countries,” CA: a Cancer Journal for Clinicians 71 (2021): 209–249, 10.3322/caac.21660. 33538338

[2] S. Gupta , W. Kong , Y. Peng , Q. Miao , and W. J. Mackillop , “Temporal Trends in the Incidence and Survival of Cancers of the Upper Aerodigestive Tract in Ontario and the United States,” International Journal of Cancer 125 (2009): 2159–2165, 10.1002/ijc.24533. 19569190

[3] C. Wang , X. Q. Liu , J. S. Hou , J. N. Wang , and H. Z. Huang , “Molecular Mechanisms of Chemoresistance in Oral Cancer,” Chinese Journal of Dental Research 19 (2016): 25–33, 10.3290/j.cjdr.a35694. 26981604

[4] S. J. Baker and E. P. Reddy , “Modulation of Life and Death by the TNF Receptor Superfamily,” Oncogene 17 (1998): 3261–3270, 10.1038/sj.onc.1202568. 9916988

[5] F. Li , G. Ambrosini , E. Y. Chu , et al., “Control of Apoptosis and Mitotic Spindle Checkpoint by Survivin,” Nature 396 (1998): 580–584, 10.1038/25141. 9859993

[6] Q. L. Deveraux , N. Roy , H. R. Stennicke , et al., “IAPs Block Apoptotic Events Induced by Caspase‐8 and Cytochrome c by Direct Inhibition of Distinct Caspases,” EMBO Journal 17 (1998): 2215–2223, 10.1093/emboj/17.8.2215. 9545235 PMC1170566

[7] B. Nachmias , Y. Ashhab , and D. Ben‐Yhuda , “The Inhibitor of Apoptosis Protein Family (IAPs): An Emerging Therapeutic Target in Cancer,” Seminars in Cancer Biology 14 (2004): 231–243, 10.1016/j.semcancer.2004.04.002. 15219616

[8] A. D. Schimmer , “Inhibitor of Apoptosis Proteins: Translating Basic Knowledge Into Clinical Practice,” Cancer Research 64 (2004): 7183–7190, 10.1158/0008-5472.CAN-04-1918. 15492230

[9] S. A. Hahn , M. Schutte , A. T. Hoque , et al., “DPC4, a Candidate Tumor Suppressor Gene at Human Chromosome 18q21.1,” Science 271 (1996): 350–353, 10.1126/science.271.5247.350. 8553070

[10] Y. Zhang , X. Feng , R. We , and R. Derynck , “Receptor‐Associated Mad Homologues Synergize as Effectors of the TGF‐Beta Response,” Nature 383 (1996): 168–172, 10.1038/383168a0. 8774881

[11] P. M. Siegel and J. Massagué , “Cytostatic and Apoptotic Actions of TGF‐Beta in Homeostasis and Cancer,” Nature Reviews Cancer 3 (2003): 807–821, 10.1038/nrc1208. 14557817

[12] L. Levy and C. S. Hill , “Alterations in Components of the TGF‐Beta Superfamily Signaling Pathways in Human Cancer,” Cytokine & Growth Factor Reviews 17 (2006): 41–58, 10.1016/j.cytogfr.2005.09.009. 16310402

[13] C. J. Dai , Y. T. Cao , F. Hung , and Y. G. Wang , “Multiple Roles of Mothers Against Decapentaplegic Homolog 4 in Tumorigenesis, Stem Cells, Drug Resistance, and Cancer Therapy,” World Journal of Stem Cells 14 (2022): 41–53, 10.4252/wjsc.v14.i1.41. 35126827 PMC8788178

[14] A. Moustaka and K. Miyazawa , TGF‐β in Human Disease (Springer, 2013), 139–168.

[15] R. Yoshida , H. Nakayama , M. Nagata , et al., “Overexpression of Nucleostemin Contributes to an Advanced Malignant Phenotype and a Poor Prognosis in Oral Squamous Cell Carcinoma,” British Journal of Cancer 111 (2014): 2308–2315, 10.1038/bjc.2014.539. 25314067 PMC4264447

[16] M. Nagata , H. Nakayama , T. Tanaka , et al., “Overexpression of cIAP2 Contributes to 5‐FU Resistance and a Poor Prognosis in Oral Squamous Cell Carcinoma,” British Journal of Cancer 105 (2011): 1322–1330, 10.1038/bjc.2011.387. 21952624 PMC3241556

[17] Y. Shimosato , S. Oboshi , and K. Baba , “Histological Evaluation of Effects of Radiotherapy and Chemotherapy for Carcinomas,” Japanese Journal of Clinical Oncology 1 (1971): 19–35, 10.1093/oxfordjournals.jjco.a039343.

[18] M. B. Amin , F. L. Greene , S. B. Edge , et al., AJCC Cancer Staging Manual, 8th ed. (Springer, 2017).

[19] L. Barnes , Pathology and Genetics of Head and Neck Tumours (IARC, 2005).

[20] J. Sakata , R. Yoshida , Y. Matsuoka , et al., “Predictive Value of the Combination of SMAD4 Expression and Lymphocyte Infiltration in Malignant Transformation of Oral Leukoplakia,” Cancer Medicine 6 (2017): 730–738, 10.1002/cam4.1005. 28256094 PMC5387127

[21] K. Yamana , J. Inoue , R. Yoshida , et al., “Extracellular Vesicles Derived From Radioresistant Oral Squamous Cell Carcinoma Contribute to the Acquisition of Radioresistance via the miR‐503‐3p‐BAK Axis,” Journal of Extracellular Vesicles 10 (2021): e12169, 10.1002/jev2.12169. 34894384 PMC8665688

[22] Y. Matsuoka , R. Yoshida , K. Kawakara , et al., “The Antioxidative Stress Regulator Nrf2 Potentiates Resioresistance of Oral Squamous Cell Carcinoma Accompanied With Metabolic Modulation,” Laboratory Investigation 102 (2022): 896–907, 10.1038/s41374-022-00776-w. 35414650 PMC9309095

[23] K. Kawahara , M. Nagata , R. Yoshida , et al., “miR‐30a Attenuates Drug Sensitivity to 5‐FU by Modulating Cell Proliferation Possibly by Downregulating Cyclin E2 in Oral Squamous Cell Carcinoma,” Biochemistry and Biophysics Reports 28 (2021): 101114, 10.1016/j.bbrep.2021.101114. 34589618 PMC8461355

[24] Y. Kuwahara , M. Mori , T. Oikawa , et al., “The Modified High‐Density Survival Assay Is the Useful Tool to Predict the Effectiveness of Fractionated Radiation Exposure,” Journal of Radiation Research 51 (2010): 297–302, 10.1269/jrr.09094. 20410675

[25] J. Sakata , A. Hirosue , R. Yoshida , et al., “Enhanced Expression of IGFBP‐3 Reduces Radiosensitivity and Is Associated With Poor Prognosis in Oral Squamous Cell Carcinoma,” Cancers 12 (2020): 494, 10.3390/cancers12020494. 32093285 PMC7072421

[26] S. Bornstein , R. White , S. Malkoski , et al., “Smad4 Loss in Mice Causes Spontaneous Head and Neck Cancer With Increased Genomic Instability and Inflammation,” Journal of Clinical Investigation 119 (2009): 3408–3419, 10.1172/JCI38854. 19841536 PMC2769185

[27] H. Cheng , E. J. Fertig , H. Ozawa , et al., “Decreased SMAD4 Expression Is Associated With Induction of Epithelial‐To‐Mesenchymal Transition and Cetuximab Resistance in Head and Neck Squamous Cell Carcinoma,” Cancer Biology & Therapy 16 (2015): 1252–1258, 10.1080/15384047.2015.1056418. 26046389 PMC4623002

[28] H. Ozawa , R. S. Ranaweera , E. Izumchenko , et al., “SMAD4 Loss Is Associated With Cetuximab Resistance and Induction of MAPK/JNK Activation in Head and Neck Cancer Cells,” Clinical Cancer Research 23 (2017): 5162–5175, 10.1158/1078-0432.CCR-16-1686. 28522603 PMC6078415

[29] J. L. Boulay , G. Mild , A. Lowy , et al., “SMAD4 Is a Predictive Marker for 5‐Fluorouracil‐Based Chemotherapy in Patients With Colorectal Cancer,” British Journal of Cancer 87 (2002): 630–634, 10.1038/sj.bjc.6600511. 12237773 PMC2364238

[30] F. D. Sun , P. C. Wang , R. L. Luan , S. H. Zhou , and X. Du , “MicroRNA‐574 Enhances Doxorubicin Resistance Through Down‐Regulating SMAD4 in Breast Cancer Cells,” European Review for Medical and Pharmacological Sciences 5 (2018): 1342–1350, 10.26355/eurrev_201803_14476. 29565492

[31] B. Zhang , B. Zhang , X. Chen , et al., “Loss of Smad4 in Colorectal Cancer Induces Resistance to 5‐Fluorouracil Through Activating Akt Pathway,” British Journal of Cancer 110 (2014): 946–957, 10.1038/bjc.2013.789. 24384683 PMC3929873

[32] S. T. Pan , Z. L. Li , Z. X. He , J. X. Qiu , and S. F. Zou , “Molecular Mechanisms for Tumour Resistance to Chemotherapy,” Clinical and Experimental Pharmacology & Physiology 43 (2016): 723–737, 10.1111/1440-1681.12581. 27097837

[33] H. S. Hofmann , A. Simm , A. Hammer , R. E. Silber , and B. Bartling , “Expression of Inhibitors of Apoptosis (IAP) Proteins in Non‐Small Cell Human Lung Cancer,” Journal of Cancer Research and Clinical Oncology 128 (2002): 554–560, 10.1007/s00432-002-0364-z. 12384799 PMC12164409

[34] H. H. Wu , J. Y. Wu , Y. W. Cheng , et al., “cIAP2 Upregulated by E6 Oncoprotein via Epidermal Growth Factor Receptor/Phosphatidylinositol 3‐Kinase/AKT Pathway Confers Resistance to Cisplatin in Human Papillomavirus 16/18‐Infected Lung Cancer,” Clinical Cancer Research 16 (2010): 5200–5210, 10.1158/1078-0432.CCR-10-0020. 20959404

[35] E. Krepela , P. Dankova , E. Moravcikova , et al., “Increased Expression of Inhibitor of Apoptosis Proteins, Survivin and XIAP, in Non‐Small Cell Lung Carcinoma,” International Journal of Oncology 35 (2009): 1449–1462, 10.3892/ijo_00000464. 19885569

[36] G. S. Salvesen and C. S. Duckett , “IAP Proteins: Blocking the Road to Death's Door,” Nature Reviews. Molecular Cell Biology 3 (2002): 401–410, 10.1038/nrm830. 12042762

[37] Y. K. Jeon , C. K. Kim , J. Koh , D. H. Chung , and G. H. Ha , “Pellino‐1 Confers Chemoresistance in Lung Cancer Cells by Upregulating cIAP2 Through Lys63‐Mediated Polyubiquitination,” Oncotarget 7 (2016): 41811–41824, 10.18632/oncotarget.9619. 27248820 PMC5173098

[38] E. W. Lee , D. Seong , J. Seo , M. Jeong , H. K. Le , and J. Song , “USP11‐Dependent Selective cIAP2 Deubiquitylation and Stabilization Determine Sensitivity to Smac Mimetics,” Cell Death and Differentiation 22 (2015): 1463–1476, 10.1038/cdd.2014.234. 25613375 PMC4532773

[39] J. Yasumoto , T. Kirita , A. Takahashi , et al., “Apoptosis‐Related Gene Expression After Hyperthermia in Human Tongue Squamous Cell Carcinoma Cells Harboring Wild‐Type or Mutated‐Type p53,” Cancer Letters 204 (2004): 41–51, 10.1016/j.canlet.2003.07.005. 14744533

[40] F. Wang , X. Xia , C. Yang , et al., “SMAD4 Gene Mutation Renders Pancreatic Cancer Resistance to Radiotherapy Through Promotion of Autophagy,” Clinical Cancer Research 24, no. 13 (2018): 3176–3185, 10.1158/1078-0432.CCR-17-3435. 29602802 PMC6345154

[41] W. Li , W. Wei , D. Hu , R. Tang , and Z. Hu , “circRNA ITGA7 Restrains Growth and Enhances Radiosensitivity by Up‐Regulating SMAD4 in Colorectal Carcinoma,” Open Medicine 18, no. 1 (2023): 20220604, 10.1515/med-2022-0604. 36694626 PMC9835197

[42] Y. Kuwahara , K. Tomita , Y. Urushihara , T. Sato , A. Kurimasa , and M. Fukumoto , “Association Between Radiation‐Induced Cell Death and Clinically Relevant Radioresistance,” Histochemistry and Cell Biology 150 (2018): 649–659, 10.1007/s00418-018-1728-z. 30232589

[43] Z. Su , Z. Yang , L. Xie , J. P. DeWitt , and Y. Chen , “Cancer Therapy in the Necroptosis Era,” Cell Death and Differentiation 23 (2016): 748–756, 10.1038/cdd.2016.8. 26915291 PMC4832112

[44] Y. Kuwahara , T. Oikawa , Y. Ochiai , et al., “Enhancement of Autophagy Is a Potential Modality for Tumors Refractory to Radiotherapy,” Cell Death & Disease 2 (2011): e177, 10.1038/cddis.2011.56. 21716292 PMC3168998

[45] D. Hanahan and R. A. Weinberg , “Hallmarks of Cancer: The Next Generation,” Cell 144 (2011): 646–674, 10.1016/j.cell.2011.02.013. 21376230

[46] P. Papageorgis , K. Cheng , S. Ozturk , et al., “Smad4 Inactivation Promotes Malignancy and Drug Resistance of Colon Cancer,” Cancer Research 71 (2011): 998–1008, 10.1158/0008-5472.CAN-09-3269. 21245094 PMC3075468

[47] X. Tang , L. Shi , N. Xie , et al., “SIRT7 Antagonizes TGF‐β Signaling and Inhibits Breast Cancer Metastasis,” Nature Communications 8 (2017): 318, 10.1038/s41467-017-00396-9. PMC5566498 28827661

[48] W. Li , D. Zhu , and S. Qin , “SIRT7 Suppresses the Epithelial‐To‐Mesenchymal Transition in Oral Squamous Cell Carcinoma Metastasis by Promoting SMAD4 Deacetylation,” Journal of Experimental & Clinical Cancer Research 37 (2018): 148, 10.1186/s13046-018-0819-y. 30001742 PMC6044017

