# Supplementary material for: SMAD4 Expression and Its Association With Treatment Resistance and Clinical Outcomes in Oral Squamous Cell Carcinoma Treated With Preoperative Chemoradiotherapy
Source: Cancer Rep (Hoboken). 2026 Aug 11;9(8):e70654. doi: 10.1002/cnr2.70654 (PMC13461526; doi:10.1002/cnr2.70654)
Supplement: Supplementary file 1 [file CNR2-9-e70654-s001.docx]

| Supplementary Table S2. List of primers used for real-time PCR | | | |
| --- | --- | --- | --- |
| *Gene* |  | Primer sequence 5'→3' |  |
| *SMAD4* | Forward | CGG ACA TTA CTG GCC T | |
|  | Reverse | TAG GGC AGC TTG AAG G | |
| *cIAP1* | Forward | ATC AGA ATT GGC AAG AGC TGG | |
|  | Reverse | TAG CAT CAT CCT TTG GTT CCC | |
| *cIAP2* | Forward | TCC TGG ATA GTC TAC TAA CTG CC | |
|  | Reverse | GCT TCT TGC AGA GAG TTT CTG AA | |
| *XIAP* | Forward | ATA GTG CCA CGC AGT CTA CAA | |
|  | Reverse | AGA TGG CCT GTC TAA GGC AAA | |
| *Survivin* | Forward | CCA GAT GAC GAC CCC ATA GAG | |
|  | Reverse | TTG TTG GTT TCC TTT GCA ATT TT | |
| *GAPDH* | Forward | CTG GGC TAC ACT GAG CAC C | |
|  | Reverse | AAG TGG TCG TTG AGG GCA ATG | |
